# High performance liquid chromatography (HPLC) based genetic diversity profiling of chilli germplasm for fruit pungency and phytochemical contents

**DOI:** 10.1186/s12870-024-05055-y

**Published:** 2024-05-07

**Authors:** Misbah Naseem, Rashid Mehmood Rana, Mahmood ul Hassan, Muhammed Azam Khan, Manal Abdulaziz Binobead, Mohamed Farouk Elsadek, Heba H. Elsalahy, Rashid Iqbal

**Affiliations:** 1 Department of Plant Breeding and Genetics, PMAS-Arid Agriculture University, Rawalpindi, 46300 Pakistan; 2 Department of Horticulture, PMAS-Arid Agriculture University, Rawalpindi, 46300 Pakistan; 3 Department of Food Science and Nutrition, College of Agriculture and Food Science, King Saud University, P.O. 2455, Riyadh, 11451 Saudi Arabia; 4 Department of Biochemistry, College of Science, King Saud University, P.O. 2455, Riyadh, 11451 Saudi Arabia; 5 Leibniz Centre for Agricultural Landscape Research (ZALF), Müncheberg, 15374 Germany; 6 Department of Agronomy, Faculty of Agriculture and Environment, The Islamia University of Bahawalpur, Bahawalpur, 63100 Pakistan

**Keywords:** PCA, Capsaicin, Total phenolic contents, Ascorbic acid, Total flavonoid contents, HPLC

## Abstract

Chilli peppers are widely consumed for their pungency, as used in flavoring the food and has many pharmaceutical and medicinal properties. Based on these properties an experiment was held using 83 varieties of chilli (Hot pepper and sweet pepper) were grown in suitable environment using Augment Block design and evaluated for fruit pungency and phytochemical contents using high proficiency liquid chromatography. Analysis of variance (ANOVA) of traits showed highly significant for all traits except for fruit length and capsaicin contents. The value of Least significant increase (LSI)was ranged 0.27-1289.9 for all traits showed high variation among varieties. Highly significant correlation was found among fruit diameter to fruit weight 0.98, while moderate to high correlation was present among all traits. The most pungent genotype 24,634 was 4.8 g in weight, while the least pungent genotypes i.e. PPE-311 (32.8 g), green wonder (40.67) had higher in weight. The genotypes 24,627, 32,344, 32,368 and 1108 marked as higher number of seeds in their placental region. It was observed that chilli genotype 24,621 had maximum length with considerable high amount of pungency act as novel cultivar. Principal component analysis (PCA) showed the high variability of 46.97 for two PCs with the eigen value 2.6 and 1.63 was recorded. Biplot analysis showed a considerable variability for fruit pungency, while huge variability was found for all traits among given varieties. PPE-311, T5 and T3 are found as highly divergent for all traits. The findings of this study are instrumental for selecting parents to improve desirable traits in future chilli pepper breeding programs. It will help plant/vegetable breeders for development of highly nutrient and pungent varieties and attractive for the consumer of food sector.

## Introduction

Chilli pepper (*Capsicum annum* L.) is known for its pungency belongs to family *Solanaceae* and the genus Capsicum. Its center of origin is South America and spread all over the world around 7000 B.C [1]. It includes 30 species out of which 5 are domesticated and used as spice and vegetables [2]. When the burning sensation in the capsaicin come in contact with the mouth mucous membrane it produces heat and spicy taste, so used frequently in foods [3]. It is also used in topical lotion and acts as pain reliever such backaches, strains, sprains, and arthritis. Beside these they are widely use in industrial purposes [4]. *Capsicum annum* L. is being used as pungent fruit types (hot pepper, American pepper, chilli, cayenne etc.) or as sweet pepper (capsicum, bell pepper, Shimla etc.) [5].

In pungent peppers of capsicum annum, the fruit pod contains compound *“capsaicin (CAP)”* which is main factor of heat followed by *“dihydro-capsaicin (DhCAP)”* collectively known as “capsaicinoids *(CAPDs)”.* Some minor capsaicinoids are also present e.g., *nordihydro-capsaicin (NDC*), *homodihydro-capsaicin (HDC)* and *homocapsaicin (HDC)* are half as potent as major capsaicinoids and measured in terms of *Scoville heat units (SHU)* [6]. The amount of capsaicinoides is 0.003-1% in total weight of plant in average [7]. Capsaicinoids comprises 90% of CAP and DhCAP and are divided into branched chain fattyacid and phenylpropanoid pathway [8]. Capsaicinoids are synthesized in epidermal tissues of placenta [9]. Beside these six capsaicinoids, relative pungency is estimated by a synthetic capsaicinoid compound *“nonivamide”* (vanillylamide of n-nonanoic acid) [10]. Vanilylamide is derived from phenylalanine through branched chain fatty acid (C9-C11), and also used to synthesize capsaicin by condensing vanilylamine [11].

Capsaicin is white crystalline in nature and has profound thermal stability with high boiling (200–210 ^0^C) and melting point (65 ^0^C), even active on boiling. It is nonpolar compound and highly soluble in ethanol and vegetable oils. But only vaguely soluble in water [12]. So liquid chromatography at high pressure (HPLC) using ethanol as mobile phase has several advantages e.g. automatic and computer-based process, advanced reproducibility, even isolate compounds can identify, short time process, quantitative determination and nondestructive process [13].

*Capsicum* spps., are mainly grown for food production with nutritional composition to support healthy food and human existence and involves wide range of natural sciences [14]. To ensure and enhance plant quality and nutritional composition, the fields of pepper metabolites are expanding [15]. To characterize the healthy compounds, mostly antioxidant properties were measured. Antioxidants have vital role in body, e.g., scavenging free radicals to protect against free radicals and prevent many diseases, also enhance and protect brain cells activity by oxidation of the essential fats in brain [16]. The polyphenol compounds (total phenolic contents, ascorbic acid and flavonoids) must be taken through diet as the human body cannot produced these compounds on their own. Phenolic contents act as scavenger of oxidative molecular oxygen, reactive oxygen species and reactive nitrogen species and act as anticancer [17].

The most reliable and popular technique is HPLC for analysis of capsaicinoids and has been associated with UV absorption detection [18], fluorescence detection [19] photodiode array and or couple with fluorescence to make it more accurate and reliable [20, 21]. In this method buffer-free elution system along with reverse phase column having conventional end-capping of C18 phase is being used to perform for separation. The total 10 min running time is enough to fractionate the pepper extract into its compound [6]. The heat levels vary widely from 0 to 500,000 Scoville heat units (SHU). They are classified as: (0–700 SHU) non-pungent; (700–3,000 SHU) mildly pungent; (25, 000–70,000 SHU) highly pungent; (3,000–25,000 SHU) moderately pungent; (> 80,000 SHU) very highly pungent [4]. Estimation of the fruit pungency and phytochemicals in the Capsicum annum among the different varieties for further genetic and inheritance studies and in breeding is the main purpose of this study.

## Materials and methods

### Plant material and experimental conditions

The research was conducted in the glasshouse and research area of Pir Mehr Ali Shah Arid Agriculture University, Rawalpindi, Pakistan. The scope of the study encompassed the evaluation of chilli plants, both spicy (hot) and non-spicy (sweet) genotypes (Table 1). A collection of 65 distinct hot chilli genotypes and 18 sweet chilli genotypes were utilized. The seeds required for the study were collected from PGRI (Plant Genetic Resource Institute), National Agriculture Research Center (NARC) Islamabad, Pakistan, and Barani Agriculture Research Institute (BARI), Chakwal, Pakistan (Table 1).

**Table 1:** Table showing the pepper type and their pungency level

Genotypes	Type of pepper	Pungency level
Loungi	Hot Pepper	High
Pusa Jawala	Hot Pepper	Moderate
1787	Hot Pepper	Moderate
1791	Hot Pepper	Moderate
1792	Hot Pepper	Moderate
1799	Hot Pepper	High
16,162	Hot Pepper	Moderate
16,163	Hot Pepper	Moderate
16,166	Hot Pepper	High
Tam	Sweet pepper	High
Scotch Bonnet	Sweet pepper	Moderate
20,366	Hot Pepper	High
20,372	Hot Pepper	High
20,451	Hot Pepper	High
20,523	Hot Pepper	Moderate
20,524	Hot Pepper	High
24,621	Hot Pepper	Moderate
24,623	Hot Pepper	High
Chocolate	Sweet pepper	Moderate
Golden	Sweet pepper	Moderate
24,626	Hot Pepper	Moderate
24,627	Hot Pepper	Moderate
24,629	Hot Pepper	Moderate
24,634	Hot Pepper	Very High
32,319	Hot Pepper	High
32,320	Hot Pepper	Moderate
32,321	Hot Pepper	High
Orange	Sweet pepper	Moderate
Purple Jalapeno	Sweet pepper	Moderate
32,330	Hot Pepper	Moderate
32,332	Hot Pepper	Moderate
32,333	Hot Pepper	Moderate
32,335	Hot Pepper	Moderate
32,336	Hot Pepper	High
32,344	Hot Pepper	Moderate
32,349	Hot Pepper	Moderate
T3	Sweet pepper	Moderate
T5	Sweet pepper	Moderate
32,354	Hot Pepper	Moderate
32,355	Hot Pepper	Very High
32,362	Hot Pepper	High
32,368	Hot Pepper	High
32,370	Hot Pepper	Moderate
32,385	Hot Pepper	Moderate
32,390	Hot Pepper	Moderate
36,111	Sweet pepper	Moderate
36,112	Sweet pepper	Mild
16/5	Hot Pepper	High
16/7	Hot Pepper	High
16/8	Hot Pepper	High
16/9	Hot Pepper	Moderate
Skyway Korea (S.Way)	Hot Pepper	Moderate
BSS-410 India	Hot Pepper	High
Skyline 2(H) Tlnd	Hot Pepper	Moderate
36,113	Sweet pepper	Moderate
36,114	Sweet pepper	Moderate
15/6	Hot Pepper	Moderate
KHHP-081 A-	Hot Pepper	Moderate
408	Hot Pepper	Moderate
1108	Hot Pepper	Moderate
1209	Hot Pepper	Moderate
9905	Hot Pepper	High
59,328	Hot Pepper	High
36,117	Sweet pepper	Mild
36,118	Sweet pepper	Moderate
Chakwal 3	Hot Pepper	Moderate
Chakwal 4	Hot Pepper	Moderate
Chakwal 5	Hot Pepper	Moderate
Ghotki	Hot Pepper	Moderate
Syngenta P-6	Hot Pepper	High
CDK	Hot Pepper	Moderate
Advanta 5017	Hot Pepper	Moderate
33,826	Sweet pepper	Mild
33,827	Sweet pepper	Mild
Green Gold	Hot Pepper	High
Super Sky	Hot Pepper	Moderate
Kalae 542 F1	Hot Pepper	High
HP 1410	Hot Pepper	Moderate
Advanta512	Hot Pepper	Moderate
PH-264 F1	Hot Pepper	Moderate
Green Fire	Hot Pepper	High
PPE-311	Sweet pepper	Mild
Green Wonder (GW)	Sweet pepper	Mild

Seeds from each genotype are treated with 3% KNO_3_ before sowing. The nursery was sown in February 2018 and 2019 using peatmoss in 98 cells plastic trays. The seedlings were nurtured for a duration of 45 days before being transplanted into an open field environment. The transplantation process was carried out using Augmented Block Designs (ABD) in April 2018 and 2019. Two designated check varieties, namely “Pusa Jawala” and “Loungi,” for comparison and reference purposes were used. In Augment Block Design, 9 blocks, each containing 9 genotypes and 2 checks with 10 plants each were used. The plants were transplanted with a plant-to-plant distance of 45 cm and row-to-row distance of 75 cm.

All recommended cultural practices viz. hoeing, weeding, irrigation, fertilization (NPK, 1:1:1) and fungicide spray were applied to control the spread of insects, pest and disease and insure healthy plant growth.

### Morphological parameters

The chilli genotypes were raised to study various quantitative parameters i.e., fruit length (FL), fruit diameter (FD), fruit length to diameter ratio (LDR), number of seeds per fruit (FS), fruit weight (FW). Fruit morphology was evaluated for the purpose of genotype categorization, employing a visual assessment conforming to the criteria required by “The World Vegetable Center (AVRDC)”.

### Phytochemical analysis

#### Preparation of plant extract

Ripen fruits were collected from the field and washed thoroughly. Seeds were removed from fruits and then separate the edible portion. and then Oven dried the fruits them at 550 C for 48 h. After drying, 10 g of dried pepper samples extracted dissolve in 100 ml distilled water (1:10) (i.e., 10 g/100 ml) to produce the aqueous extracts. After that placed aqueous extracts in ultrasonic instrument for 30 min, left up to 24 h at 15 °C filter it through a Whatman paper No. 1.

#### Determination of Total Flavonoid Contents (TFC)

For sample preparation 500 µl of extract was mixed in 2 ml of distilled water to make a solution. 150 µl of 5% sodium nitrate was added in this solution. Refluxed the solution for 5 min and then added 150 µl of 10% AlCl_3_ and 2000 µl of 1 M sodium hydroxide. Again added 1200 µl of distilled water in given solution. The solution was incubated for 30 min.

The prepared solution was than centrifuged at 10,000 rpm and filter through 0.2 μm Milipore membrane filter. Remove the supernatant. 1–3 ml was collected in syringe and injected in HPLC Agilent (Series 1200). Fluorometric detection was carried out at wavelength of 330 nm. Peaks were detected for each sample using ultraviolet detector (310 nm) through using HP software. Flavonoids contents were measure using quercetin as standard. Linearity was found to be in the range of 2 to 10 µg/ml. Standard solution was prepared from a stock solution of quercetin using five dilutions 2, 4, 6, 8 and 10 µg/ml. Each concentration was injected trice and average was found and present in Table 2.

**Table 2:** HPLC analysis showing different parameters for Total Flavonoid contents, Total phenolic contents, Ascorbic acid, Capsaicin and Dihydrocapsaicin

Components	LOD (µg/g)	LOQ (µg/g)	Linearity range (µg/ml)	Linearity curve	Retention time (min)	*R*^2^
Total flavonoid contents	126	382	2-8	Y = 0.297x + 0.5712	2.6	0.94
Total Phenolic contents	3.53 × 10^−06^	1.07 × 10^−05^	0-1	Y = 354597x + 13,311	3.7	0.95
Ascorbic acid	6.3 × 10^−05^	1.93 × 10^−05^	1-6	Y = 300220x	2.8	0.98
Capsaicin	21	60	0-800	Y = 50.681x-875.55	2.79	0.997
Dihydrocapsaicin	40	130	0-800	Y = 25.494x-113.85	3.5	0.999

#### Determination of Total Phenolic Contents (TPC)

Folin Ciocalteu reagent was prepared by dissolving 10 g of sodium tungstate and 2.5 g of sodium molybdate in 70 ml of water. After that 5 ml of 85% phosphoric acid and 10 ml of concentrated hydrochloric acid was added. The mixture is then subjected to reflux for at least 10 h. Then add 15 g of lithium sulfate, 5 ml of water, and 1 drop of bromine and Reflux for 15 min. Cool to room temperature and bring to 100 ml with water. The reaction mixture was contain 1 ml of extract, 0.5 ml of the Folin-Ciocalteu reagent, 1 ml sodium carbonate 7.5% and 7.5 ml of distilled water was added, respectively. After 45 min of reaction at ambient temperature, using a UV-visible spectrophotometer measure the absorbance at 280 nm. Total phenolic content of samples were determined in triplicates and the results will be expressed on dry weight basis (DW) as mg gallic acid equivalents (GAE), per g of each sample [22]. For calculation of linearity the six concentrations of standard gallic acid ranged 0 to 1 µg/g were used. The calibration curve was obtained by using standards 0, 0.0625, 0.12, 0.25 and 0.5 µg/g.

The aqueous extracts were centrifuged at 10 000 rpm for 10 min and the supernatant was filtrated through a 0.2 μm millipore membrane filter. Then 1–3 ml was be collected in a vial for injection into HPLC Agilent (Series 1200) equipped with auto sampler injector, solvent degasser, ultraviolet (UV) detector set at 280 nm and quaternary HP pump (Series 1100). The column [Agilent 5HC-C18 (2) 250 × 4.6 mm] temperature was maintained at 35 °C. Gradient separation was carried out with methanol and acetonitrile as a mobile phase at flow rate of 1 ml/min. Phenolic acid standards from sigma Co. was dissolved in a mobile phase and injected into HPLC. Retention time and peak area of the tested samples were calibrated against standard solutions of different phenolic and aromatic compounds concentration by the data analysis.

#### Determination of Ascorbic Acid (ASCA)

One g of dried pepper samples mixed with 0.3% metaphosphoric acid solution and centrifuged at 10 000 rpm for 10 min. The supernatant wase filtrated through a 0.2 μm Millipore membrane filter. Then 1–3 ml was collected in a vial for injection into HPLC Agilent (Series 1200) equipped with auto sampler injector, solvent degasser, ultraviolet (UV) detector (set at 254 nm) and quaternary HP pump (Series 3365). The column [Agilent 5HC-C18 (2) 250 × 4.6 mm] temperature was maintained at 25 °C. Ascorbic acid was identified by comparing the retention time of the sample peak with that of the ascorbic standard at 280 nm [23]. The concentration of standard used ranged from 1 to 6 mg/g.

#### Capsaicin contents (PUN)

The fruit pungency was measured at ripping stage by using standard HPLC protocol. Three grams of well-blended pepper sample crushed in a crucible mortar with quartz sand, in the macerate, 50 ml of methanol (analytical grade) was added and then transfer the mixture to a 100 ml erlenmeyer flask. The mixture wias subjected to 4 min long ultra-sonication. Then filtered through filter paper. The filtrate was more purified by passing through a 0.45 mm PTFE syringe filter before injection on the HPLC column. After suitable dilution, the extract injected to Nucleodur C18, Cross-Linked. The separation was performed with isocratic elution of 50:50 water-acetonitrile and a flow rate of 0.8 ml/min. Fluorometric detection of capsaicinoid carried out at wavelength 280 nm [24].

### Statistical analysis

The data were subjected to analysis of variance (ANOVA), principal component analysis (PCA) and Pearson’s correlation using R-project (v. 3.3.1). HPLC parameters along with the calibration cure were generated using the data obtained. Fruit pungency was calculated in SHU by using the given formula:

$$\text{S}\text{H}\text{U} = \left(\text{c}\text{a}\text{p}\text{s}\text{a}\text{i}\text{c}\text{i}\text{n} \times 16.1\right)+ \left(\text{d}\text{i}\text{h}\text{y}\text{d}\text{r}\text{o}\text{c}\text{a}\text{p}\text{s}\text{a}\text{i}\text{c}\text{i}\text{n} \times 16.1\right)$$

## Results

### HPLC profiling

#### Total Flavonoid Contents (TFC)

**Fig. 1:**
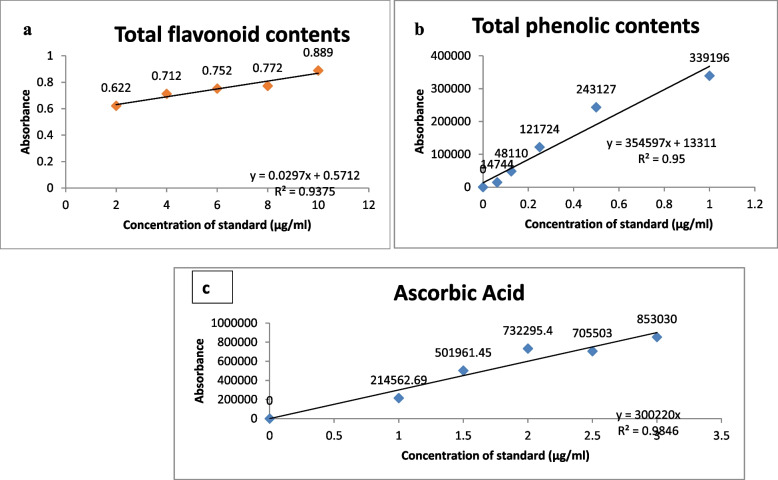
Calibration curve, (**a**) regression equation for total flavonoid contents, (**b**) regression equation for total phenolic contents, (**c**) regression equation for ascorbic acid

The standard regression equation was derived from calibration curve by plotting peak area against the concentration is *Y = 0.0297x + 0.5712* with the regression constant (*R*^2^) is 0.94, with the retention time. The value of *R*^2^ was significant validate the good linearity of the method (Fig. 1a). The limit of detection (LOD) was measured as 126 µg/g, while the limit of quantification (LOQ) was measured for total flavonoid contents was measured as 382 µg/g (Table 2).

#### Total Phenolic Contents (TPC)

The regression equation was derived as *Y = 354597x + 13311*with the *R*² = 0.95. The value of *R*^2^ is highly significant indicating high linearity and validation of the method used (Fig. 1b). The limit of detection (LOD) and limit of quantification was measured as was estimated as 3.53 × 10^−06^ µg/kg and 1.07 × 10^−05^ µg/kg respectively (Table 2).

#### Ascorbic acid (ASCA)

The linearity was found using the calibration curve and obtained standard regression equation as *Y = 300220x* with the regression constant R2 as 0.98, which showed the high linearity for the method we used (Fig. 1c). The value of limit of detection (LOD) was measured as 6.38 × 10^−06^ and the limit of quantification (LOQ) was measured as 1.93 × 10^−05^ (Table 2).

#### Capsaicin contents (PUN)

**Fig. 2:**
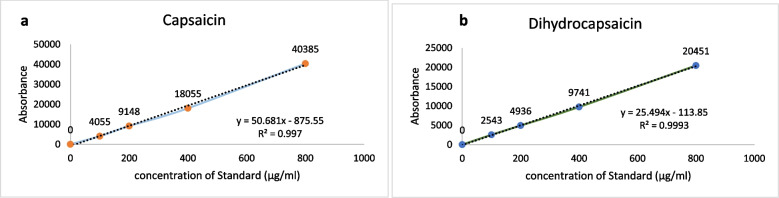
Calibration curve, (**a**) regression equation for Capsaicin, (**b**) regression equation for Dihydrocapsaicin

The regression equation was estimated as *Y = 50.681x-875.55* and *Y = 25.494x-113.85* respectively for capsaicin and dihydrocapsaicin. The average retention time for capsaicin was recorded as 2.79 and 3.5 for dihyrocapsaicin. The *R*^2^ was found as 0.997 and 0.999 for capsaicin and dihydrocapsaicin respectively, which is highly significant and validate the authenticity of the method used (Fig. 2a and b). The linearity was found very effective as value of limit of detection (LOD) and limit of quantification (LOQ) was calculated as 21 µg/g and 60 µg/g found to be highly linear for capsaicin. For dihydrocapsaicin the value of limit of detection (LOD) was measured as 40 µg/g and limit of quantification (LOQ) as 130 µg/g (Table 2). Several studies indicated that capsaicin is not a simple factor and varies in chilli fruit varieties and depend on many internal compositions of genetic factors of different varieties, environment and antioxidants. The activity of enzymes capsaicin syntheses and peroxidase has profound effect on pungency [25].

#### Analysis of variance

Fruit pungency is most important character with respect to breeding chilli. It is most vital to increase pungency for enhancement of fruit quality and other quality parameters are directly and indirectly related to the chilli. To find the diversity among chilli genotypes 83 genotypes are collected from different sources (Table 3) and studied for 4 quality traits using augment block design and HPLC for measuring fruit pungency, fruit flavonoids contents, fruit phenolic contents and fruit ascorbic acid at maturity stage. Analysis of variance showed that there is no significant differences are present among blocks for all traits under studies and hence improvement within blocks is not possible in this case. Significance level is highest up to *p* < 0.01 in control for all traits except fruit diameter and fruit weight. Augment showed the high significant results up to *p* < 0.01 for all traits except fruit pungency and fruit length, same trend was observed for control vs. augment. Overall results explain the higher diversity level and chances of improvement using this population under study. Coefficient of variation ranges from 5.6 to 69.2% for all traits under study (Table 3).

**Table 3:** Means of the parameter under study

Genotype	Flavonoids (ppm)	Phenolic contents (ppm)	Vitamin C. (ppm)	Pungency (SHU)	Fruit length (dm)	Fruit diameter (dm)	Fruit length to diameter ratio	Number of seeds/fruit	Fruit weight (g)
Loungi	10.330	2.159	1.109	32156.02	40	51	0.8	53	3.5
Pusa Jawala	3.057	0.331	22.576	9995.497	126	32	3.9	38	4
1787	6.760	0.378	0.007	10518.27	43	4.2	10.2	65	3.7797
1791	5.785	2.707	1.509	10160.93	39	7.9	4.9	33	4.395
1792	6.492	4.048	1.161	6727.255	88	8.9	9.9	40	3.0765
1799	1.407	0.095	0.846	30995.19	32	10.3	3.1	46	3.4281
16,162	1.778	7.475	1.610	16086.97	61.6	15.1	4.1	49	3.4281
16,163	10.330	2.159	1.109	10054.98	33.6	15.5	2.2	46	2.637
16,166	2.110	6.051	0.207	31021.63	75	7.1	10.6	31	1.9338
TAM	7.569	4.437	0.152	26798.2	63.3	76.7	1.2	21	15
Scotch Bonnet	5.549	3.264	1.309	7602.176	54	104.6	1.9	11	4.67
20,366	5.550	1.022	12.316	55995.85	44	11.5	3.8	37	5.274
20,372	5.616	3.778	0.001	21209.17	39.6	14.7	2.7	35	3.1644
20,451	7.670	6.417	0.220	33006.53	63	11.3	5.6	68	3.0765
20,523	6.896	0.158	0.001	19681.55	52	10.6	4.9	37	2.0217
20,524	7.468	2.934	1.385	25493.63	29	20.6	1.4	52	2.4612
24,621	3.057	0.331	22.576	21306.8	149.5	14.5	8.9	32	3.6039
24,623	5.414	6.849	1.766	26,999	31	43.7	0.7	65	4.8345
Chocolate	2.121	1.321	0.174	6832.374	18.6	34.67	1.9	21	4
Golden	4.111	3.872	2.134	7363.842	114	112	1.0	17	23
24,626	1.205	1.048	2.389	20178.05	78	8	9.8	49	3.9555
24,627	6.222	1.241	5.765	10200.97	78	19.3	4.0	82	2.8128
24,629	2.754	12.491	1.717	24639.09	25	13.8	1.8	50	3.516
24,634	8.242	2.262	2.620	95007.18	44	15.2	2.9	63	4.8345
32,319	3.562	4.783	1.200	69580.73	46	18.9	2.4	62	2.7249
32,320	2.081	4.317	4.086	15991.05	80	10.7	7.5	48	4.2192
32,321	3.630	4.503	5.656	25018.27	49	14	3.5	67	2.9007
Orange	5.031	5.876	2.011	5736.084	82	150.6	1.8	18	20.33
Purple Jalapeno	1.872	4.653	1.863	3637.057	58	74.6	1.3	25	5.67
32,330	6.727	0.391	2.860	6856.08	78	11.5	6.8	39	3.3402
32,332	5.650	0.011	10.995	23,381	34	13.9	2.4	31	4.395
32,333	6.121	5.404	0.115	11919.45	35	13.7	2.6	55	5.0103
32,335	7.064	7.381	0.327	12780.43	50	10.2	4.9	45	5.4498
32,336	4.572	4.987	9.293	30810.35	62.5	12.8	4.9	52	5.7135
32,344	7.350	6.140	0.768	17889.81	40	18.4	2.2	92	5.3619
32,349	5.481	0.327	37.657	20026.67	87	9.3	9.4	48	4.5708
T3	3.980	2.763	1.986	3120.997	78.7	150.6	1.9	23	52
T5	1.097	3.981	2.761	7209.008	63.3	175	2.8	26	31
32,354	2.013	3.195	6.246	24505.69	82.5	9.8	8.4	15	3.1644
32,355	5.380	1.540	0.482	104572.2	41	15.5	2.6	39	3.2523
32,362	7.333	3.596	5.363	51384.25	32	8.2	3.9	23	3.7797
32,368	5.886	5.152	18.807	59558.1	46	16.8	2.7	90	4.0434
32,370	5.313	0.315	1.108	14944.38	49.5	9.4	5.3	41	4.2192
32,385	4.471	2.664	1.599	18544.44	100	13.1	7.6	56	4.1313
32,390	8.478	1.473	1.936	15181.52	69	13.5	5.1	44	5.0103
36,111	2.985	1.873	1.097	5662.193	22	23.67	1.1	20	3.33
36,112	1.987	0.871	0.654	2717.266	18	33.67	1.9	23	3
16/5	5.582	0.157	4.244	26513.21	96	10.4	9.2	47	3.6918
16/7	5.582	1.402	0.428	25662.22	49.5	13.1	3.8	25	1.4943
16/8	6.929	1.003	3.266	26328.55	128	15.9	8.1	59	4.9224
16/9	9.589	0.271	0.601	14062.9	53	13	4.1	29	4.395
Skyway Korea	7.098	1.700	0.631	23053.71	67	8.2	8.2	26	4.6587
BSS-410 India	10.970	0.596	0.406	57003.3	61.5	8.6	7.2	35	2.2854
SkyLine 2(H) Tlnd	5.818	0.438	0.617	18156.54	57.5	10.2	5.6	41	3.7797
36,113	2.563	1.935	2.431	6668.455	97	24.3	2.5	21	4.67
36,114	1.092	1.001	2.130	2243.298	25	50.7	2.0	20	2.33
15/6	6.088	1.230	8.496	6227.457	34	17	2.0	44	4.4829
KHHP-081 A-	9.690	4.274	2.938	14788.48	117.5	19.2	6.1	41	3.3402
408	9.657	1.410	0.114	6788.183	82.5	12.8	6.4	52	3.8676
1108	9.017	9.526	3.949	12075.7	46	21	2.2	92	4.0434
1209	8.815	3.287	3.536	13477.36	81	39	2.1	61	4.8345
9905	6.424	0.082	11.442	30428.67	29	52	0.6	57	4.3071
59,328	7.098	7.314	1.847	42749.32	55	38	1.4	71	8.0868
36,117	1.561	2.011	1.054	2770.667	38.67	59.67	1.5	21	6.3
36,118	2.671	2.102	1.891	4598.051	27.6	48.33	1.8	13	7.67
Chakwal 3	6.424	1.821	0.001	16507.46	74	51	1.5	59	5.98
Chakwal 4	10.801	12.847	19.359	7897.028	51	49	1.0	48	5.3619
Chakwal 5	7.064	3.503	2.198	22548.41	77	51	1.5	54	4.4829
Ghotki	7.737	2.154	1.116	9986.566	68	43	1.6	55	5.0982
Syngenta P-6	8.276	2.198	7.525	32366.01	81	46	1.8	13	1.2
cdk	5.987	12.400	1.407	8531.98	57	26	2.2	17	5.91
Advanta 5017	7.434	1.774	0.557	13324.01	105	35	3.0	11	4.1
33,826	2.834	1.901	2.110	2153.755	32	47.6	1.5	10	16.67
33,827	1.052	2.103	0.006	2867.925	23	16	0.7	6	16
Green Gold	7.737	2.144	1.106	63371.72	69	35	2.0	29	5.87
Super Sky	7.333	0.341	22.432	18316.98	59	49	1.2	21	5.32
Kalae 542 F1	7.434	0.330	22.398	42697.84	84	32	2.6	24	5.2
HP 1410	5.448	13.878	2.627	7073.269	123	46	2.7	22	6.4
Advanta512	11.037	12.914	19.433	8619.484	79	43	1.8	14	5.89
PH-264 F1	7.569	4.437	0.152	6844.441	74	43	1.7	26	7.4
Green Fire	5.549	3.264	1.309	33435.98	93	49	1.9	33	6.1
PPE-311	11.037	12.934	19.433	2231.132	75	180	2.4	18	32.8
Green Wonder (GW)	6.054	3.192	0.103	2631.897	105	187	1.8	28	40.67

Total flavonoid contents have great impact on the pungency and observed the range from maximum (11.037 ppm) in Advanta-512 and minimum for ORANGE (1.031ppm), the average mean was 5.78 ppm. The LSI calculated for flavonoid contents was 0.339, hence the genotypes showed mean value over 10.669 ppm was considered as significant, which are only three in total. At maturity stage the most active compounds are capsaicin and phenolic contents and ascorbic acid. Total phenolic contents showed the range from maximum in HP-1410 (13.87ppm) while the minimum contents are present in line with accession number 32,332 (0.011ppm) with the mean value 4.2 ppm. The LSI calculated for phenolic compounds was 0.034, hence significant mean value should be 2.19 ppm. Thirty-nine genotypes showed higher mean value hence indicate wide range of improvement for phenolic contents in chilli. In phytochemicals, ascorbic acid range was calculated (19.433ppm) as maximum in Advanta-512 and minimum (0.007ppm) in line with accession number 1787 with average mean was calculated as 5.6 ppm. The LSI was calculated 0.33, hence the mean significant value 22.9 ppm. Only one line with accession number 32,349 showed significant higher value. Capsaicin contents was measured in terms of SHU and have range from maximum (95007.18 SHU) in 24634to minimum (2153.75) in a line with accession number 33,826, with the mean value of 21816.57 SHU. The LSI was calculated as 1289.9, hence least significant value 33445.94 SHU, none of genotype showed significant valve.

Shape and size have great variation among Capsicum species and widely used in breeding programs, as the size has direct impact on pungency. Fruit shape and size are explained in terms of fruit length to diameter ratio and observed highest in line with the accession number 16,166 (10.6) and lowest in line with accession number 24,621 (0.7) with the average of 2.66. The LSI was calculated as 0.334 and significant mean was 4.27. Twenty-two genotypes showed the maximum value than significant mean, hence provide great breeding material for future. Number of seeds per fruit and fruit weight is also important in determination of fruit size. Number of seeds per fruit was recorded highest in line with accession number 32,344 and 1108 (92 seeds per fruit), while lowest count is in line with accession number 33,827 (6 seeds per fruit) with the average of 46.87 seeds per fruit. LSI was calculated as 58.07 and significant mean was 111.08 seeds per fruit. Again, none of genotype performs better than this. Fruit weight was calculated at immature stage and observed high in variety T3 (52 g) while observed least in variety CDK (1.2 g) with the average of 6.3 g. The LSI was calculated as 0.27 and significant mean was calculated as 4.27 g., total of forty-three genotypes showed higher mean explaining the better potential for improving fruit quality in chilli (Table 4).

**Table 4:** Table showing the analysis of variance of the parameter under study

**Source of variation**	**Blocks**	**Control**	**Augment**	**Control vs**. **augment**	**Error**	**Range**	**Mean**	**CV%**	**LSI**
Flavonoids contents	0.26NS	225.1***	7.3***	9.9**	0.6	1.03-11.04	5.8	13.4	0.34
Phenolic contents	0.13NS	14.04***	206.83***	141.73***	0.06	0.11-13.87	4.2	5.6	0.34
Vitamin C.	0.54NS	1717.98***	47.84***	730.7***	0.58	0.007-19.43	5.6	13.5	0.33
Capsaicin content (MSHU)	2325.2NS	18719.2*	3891.4NS	2715.0NS	2279.1	2153.75-32156.2	21816.57	69.2	1289.9
Fruit length	4.58NS	228.7***	5.1NS	7.13NS	2.45	18-129.5	5.96	26.3	1.39
Fruit diameter	1.2NS	0.07NS	12.4***	0.59NS	0.89	4.2-187	3.9	24.3	0.51
Fruit length to diameter ratio	0.82NS	22.43***	2.23*	12.57**	0.59	0.7-10.6	2.66	28.9	0.334
Number of seed/fruit	208.8	3872***	550.5*	3186.7***	149.7	6-92	46.87	21.6	58.07
Fruit weight	0.97	0.95NS	47.7***	72.1***	0.48	1.2-52	6.3	11	0.27

#### Pearson correlation

Pearson correlation was measured for all traits to find the interrelation between these traits. TF, TP and ascorbic acid has direct relation with the antioxidant activity and active at maturity stage. Negative correlation was found between capsaicin to TP and ascorbic acid. The interrelation between these traits is also negligible. This will be explained as the effect of heat, as the TP and ascorbic acid were greatly reducing with the increase in temperature [26]. There was no significant relationship between TF, TP, Ascorbic acid to FL, FD, LDR, FW and number of seeds per fruit and found negative for most of the traits. Except for FL all fruit size related traits, FD, LDR, FW and number of seeds per fruit was found significantly correlated with capsaicin contents at *p* < 0.5. The correlation is positively highest for capsaicin contents and number of seeds per fruit and recorded as 0.59. This was explained as the accumulation of capsaicin is higher in placental region so greater number of seeds more the placental area and more the pungency. Highest correlation was found between FD and FW (*r* = 0.98, *p* < 0.01) and was highly significant. Correlation was found highly negative but significant at *p* < 0.5 for FS and FW, refers as higher the size lowers the number of seed per fruit. There is high positive interrelation was observed between FL and LDR (0.45), showed that LDR was depended on FL rather than FD, where the correlation was observed high but negative (*r* =-0.74, *p* < 0.5) (Table 5).

**Table 5:**
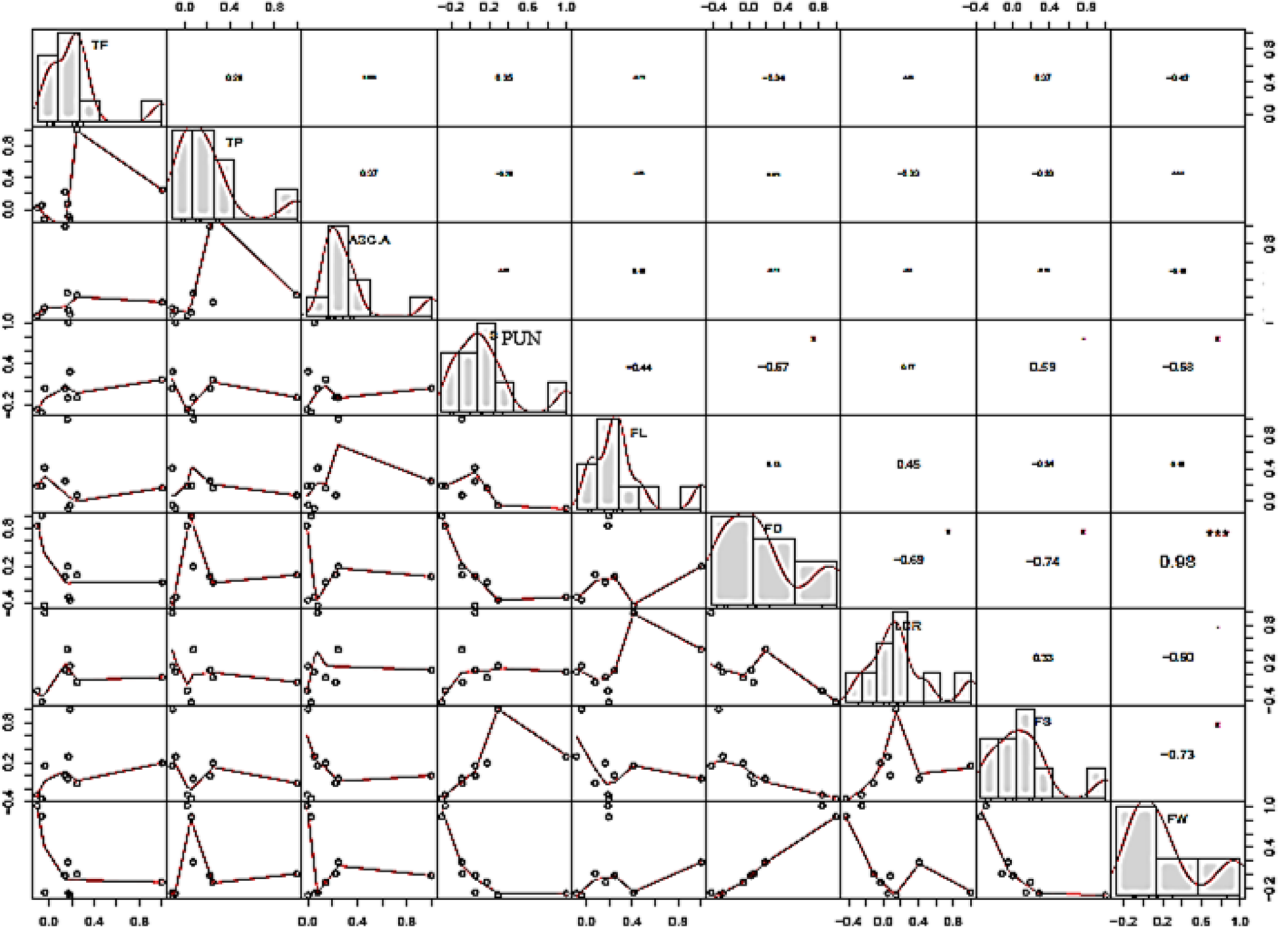
Table showing the correlation of traits under study

#### Principal component analysis

**Fig. 3:**
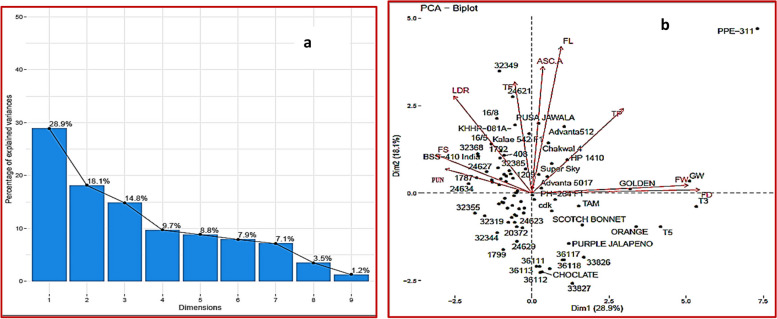
**a **Screen Plot of traits under study (**b**) Biplot for traits under study

The multivariate analysis method also known as principal component analysis (PCA) is a simple way to extract required information from large set of data and also applied for assessing genetic diversity [7]. The role of eigen values is to quantify the individual component to the gross variance and each individual eigen vector depicts the percent contribution of a variable to its respective PC [8, 14]. The eigen values indicate percentage contribution of the individual component in respect to PC. Over all two PCs have cumulative frequency estimated as 46.97 with the eigen value > 1. The variation of PC1 was recorded highest as 28.87%. Loading value of each variable was also calculated, loading value more than 0.5 or more are considered as major contributor and positive and negative signs showed direction on plot. For PC1 the major contributors for different traits are TF (-0.95), TP (0.51), PUN (-0.5), FD (0.93), FS (-0.53) and FW (0.86). The primary component revealed a significant portion of overall variability (28.87%). This was predominantly influenced by several traits exhibiting high positive values such as total phenolic contents, fruit diameter and fruit weight, alongside traits with lower positive values including ascorbic acid and fruit length (Refer to Table 5). The variation within this component predominantly related to quantitative fruit length and fruit weight characteristics and quantitative in nature. Upon examining the correlation matrix of these traits, it was evident that fruit attributes within this component exhibited notable positive correlations with total flavonoid content and total phenolic contents. This suggests a potential complementarity among these characteristics, indicating their significance in morphological characterization across the chilli genotypes. Conversely, significant negative values within this component were observed for total flavonoid contents, fruit pungency, number of seeds per fruit and fruit length to diameter ratio. The promising genotypes in PC1 based on screen plot (Fig. 3) were green wonder, T3, T5, Golden, orange, 33,826, and PPE-311 showed highly diverse for the major contributor in this PC (Table 6).

**Table 6:** Principal component analysis of traits under study

Components	PC1	PC2
EV	2.6	1.63
V (%)	28.87	18.1
C (%)	28.87	46.97
TF	-0.095	0.054
TP	0.51	0.41
ASCA	0.06	0.62
PUN	-0.5	0.12
FL	0.16	0.7
FD	0.93	0.016
LDR	-0.43	0.47
FS	-0.53	0.19
FW	0.86	0.04

*PC* Principal component, *E.V* Eigen Value, *V(%)* Variability, *C(%)* Cumulative variance, *TF* Total Flavonoid contents, *TP* Total Phenolic contents, *ASCA* Ascorbic Acid, *FL* Fruit Length, *FD* Fruit Diameter, *LDR* Fruit Length to diameter Ratio, *FS* Number of Seeds/Fruit, *FW* Fruit Weight, *PUN* Capsaicin contents

Approximately 18.1% of the variability was attributed to the second principal component. For PC2 the major contributors are ASC.A (0.62) and FL (0.7) (Table 6). These variables TF, TP, PUN, FD, FS, FW are having same values are considered as correlated and complement to each other, while in PC2 ASC.A and FL are compliment to each other and mostly quantitative in nature. This component displayed high positive values for total phenolic contents, ascorbic acid, fruit length and length to diameter ratio, while exhibiting low positive values for total flavonoid contents, fruit pungency, fruit diameter, fruit weight and number of seeds per fruit. Conversely, no negative values were observed. The correlation matrix further indicated a notable positive correlation among all traits. This suggests a potential synergy among these traits, thereby highlighting their importance in crop enhancement efforts. The genotypes BSS-414, 32,349, 16/5 and KHHP-081-A showed diversity within PC2, and these genotypes showed highly diverse for the length to diameter ratio, number of seed per fruit, fruit pungency and total flavonoid contents (Table 6).

These scores have the potential to formulate specific selection indices, with their weighting determined by the variability explained by each principal component. A high score in a particular principal component for a genotype indicates elevated values for the variables associated with that genotype within that specific component. Genotypes PPE-311, 32,349, Green wonder, 33,827 and chocolate are far away from the center in biplot, showed the diverse nature and diversity among genotypes (Fig. 3a and b). These mostly belongs to bell pepper group having difference in pungency, fruit diameter, fruit weight, fruit length to diameter ratio and number of seeds per fruit which also correlate to total phenolic contents, total flavonoid contents, hence from diverse group in this study.

## Discussions

The significant breeding required the availability of diversity in crop plants for required traits. A collection of 83 genotypes were used in this experiment were mostly used by local farmers or accessions under the process of variety development. On an international level, this collection constitutes a valuable reservoir of useful diversity within a cultivated context, which could be readily utilized for breeding objectives. To achieve this objective, the precise evaluation and documentation of trait variations are critically important for the success of any initiative focused on selecting genotypes that possess characteristics highly sought after by consumers. Likewise, the assessment of genetic diversity among genotypes is crucial not only for the preservation and safeguarding of genetic resources but also for expanding the genetic foundation of cultivated varieties and fostering sustainable agriculture [27]. In order to have better knowledge on chilli landraces yield and their nutritional aspects both approaches were addressed in this paper.

Agronomic results obtained from phenotypic and phytochemical evaluation of genotypes used under study indicate the considerable degree of divergence between the genotypes. Analysis of variance indicates the significant differences among 83 genotypes for all traits under study. Significance level is highest up to *p* < 0.01 in control for all traits except fruit diameter and fruit weight. Augment showed the high significant results up to *p* < 0.01 for all traits except fruit pungency and fruit length. Overall results explain the higher diversity level with coefficient of variation ranges from 5.6 to 69.2% for all traits under study. Extensive genetic variability in numerous traits has been investigated in chili studies [1, 10, 15].

The great variability in chili fruit length explains the great diversity present among the chili genotypes and explains that fruit shape is greatly affected by the length of the fruit. Fruit shape is greatly affected by fruit diameter. It was observed that chilli genotype 24,621 had maximum length with considerable high amount of pungency act as novel cultivar. This may be due to smaller fruit diameter and fleshy nature due to large placental region and higher weight [28]. The fruit length to diameter ratio is indicator of both fruit shape and size estimation. A larger fruit diameter results in small fruit size, resulting in a compacted and distinct fruit appearance. Typically, the fruit’s length exerts a more effect on its shape, consequently directly impacting the ratio. The principle that greater length leads to a proportionally greater ratio holds true [29]. Number of seeds per fruit directly correlated to the fruit size and shape and depends on fruit length and fruit diameter. It was estimated that the smaller fruits have more seeds per fruit as seeds only found in placental region. The genotypes 24,627, 32,344, 32,368 and 1108 marked as higher number of seeds in their placental region. These genotypes also had higher phytochemicals indicating the relation among these traits. It also depends on the pungency of the fruit, sweet or bell pepper has a lesser number of seeds due to lesser capsaicin contents in it [25]. It is evident from the population study the divergent behavior of fruit weight.

Yield has no impact all alone unless it has some supportive traits. The fruit weight observed in our study has negative impact on fruit pungency. The most pungent genotype has 24,634 was 4.8 g in weight, while the least pungent genotypes i.e. PPE-311, Green wonder, purple jalapeno had higher in weight because they are sweet chilli genotypes. Selection would be better for the development of hybrids by the presence of great variability [30]. The significant variations observed in yield components and fruit characteristics (as presented in Tables 3 and 4) indicate the strong emphasis placed on selecting for parameters related to yield and fruit quality. This parallels the scenario observed in New Mexico pepper landraces [12]. These landraces exhibited heterogeneity and endured extensive farmer-driven selection for diverse traits, ultimately adapting to local conditions through natural selection.

In vegetables there are more than 7000 different kinds of flavonoids have been found of which the primary is quercetin, luteoline, kaempferol, catechin, epicatechin, epicatechin etc [17]. . The variation is due to these different kinds of flavonoids and their concentration present in genotype. It also depends on the land races, time of ripening, the stage of chilli fruit, and the way to extract and measure them. Further this trait also depends on the structure and greatly influenced the antioxidant activity. Total phenolic content varied greatly in different genotypes, showed it to be highly variable parameter. Several studies showed that placental region have more phenolic contents than other, so it also highly related to the pungency of genotype. There is greater scope of enhance this trait using the more variability [1].

Ascorbic acid is important antioxidant and variability was present not only in genotype but within the plants of same genotype. The reason of variability may be the difference in the origin and region of cultivation of crop [29]. The environmental conditions may not only be the sole reason of variation, but also other factor may contribute to the variation like antioxidants i.e. phenolic contents and flavonoid contents. So, there is need to evaluate the ascorbic acid while considering all possible causes [30]. Several studies indicated that capsaicin is not a simple factor and varies in chilli fruit varieties and depend on many internal compositions of genetic factors of different varieties, environment and antioxidants. Also the activity of enzymes capsaicin synthase and peroxidase has profound effect on pungency [31]. Globally, the agro-morphological characterization conducted here has furnished valuable insights for farmers and breeders, aiding in the identification of well-defined horticultural types and facilitating the selection of varieties that are better suited for specific purposes. The collection exhibits both the uniformity of chili genotypes and the presence of uncommon types, such as landraces featuring large, fleshy, and pungent fruits (i.e., Kalae 542 F1, 24,621, and Syngenta P-6), as well as those with small, non-pungent berries (36,114, 36,112, 36,111). Additionally, several landraces contain noteworthy concentrations of nutraceutical compounds (i.e., 32,349), offering opportunities to develop pepper varieties with enhanced value and explore innovative market niches in the near term.

Correlation was found to be highly negative but significant at *p* < 0.5 for number of seed per fruit and fruit weight, refers as higher the size lowers the number of seeds per fruit. There is a high positive correlation was observed between fruit length and fruit length to diameter ratio, showed that LDR was depended on fruit length rather than fruit diameter. The highest correlation was observed with *r* = 0.98 between FW and FD at *p* < 0.01 (Fig. 2). This showed that all these traits are highly correlated and have positive effect on the yield [32]. The notably significant correlations identified between fruit weight and fruit diameter align with findings from various prior studies in the field of pepper research [4]. Accessions displaying higher fruit weight may accumulate low levels of capsaicinoids, given that these compounds are synthesized in the placental tissue [33]. Enhancing fruit weight contributes to the degradation of a number of placental cells, thereby leading to the accumulation of lower levels of capsaicinoids responsible for the pungent taste [31]. According to the observed correlations, three characteristics—number of seed per fruit, fruit weight, and fruit length—play a significant role in determining yield.

A Screen Biplot, depicted in Fig. 3b, elucidated the percentage of variance associated with eigen values and principal components (PCs) by graphing each PC. PC1 revealed the highest variation at 28.9%, with an eigen value of 2.6, gradually diminishing in subsequent principal components. A semi-curved line was observed, which, after the fifth PC, tended to straighten with minimal variance in each PC. The graph indicates that PC1 exhibited the maximum variation compared to the other four PCs, making the selection of lines for traits under PC1 desirable. The principal coordinates biplots (Fig. 3a and b) illustrated that more than 46.9% of the variation among genotypes, based on quantitative data from two sites, was explained by the first two PCs. This finding supports the results of the analysis of variance. Among the quantitative traits, fruit length and fruit weight had the most significant contributions to genotype organization. However, in this context, distinct genotypes from specific landraces were scattered more widely. This dispersion might be attributed not only to the influence of other characters but also to the presence of certain intermediate morphotypes among different landraces that are challenging to classify based on visual inspection [4].

These variables with almost similar values are considered as correlated and complement each other and mostly quantitative in nature. Genotypes PPE-311, 32,349, Green wonder, 33,827 and Chocolate are far away from the center in Biplot, showed the diverse nature and diversity among genotypes (Fig. 2). These mostly belongs to bell pepper group having difference in pungency, fruit diameter, fruit weight, fruit length to diameter ratio and number of seeds per fruit which also correlate to total phenolic contents, total flavonoid contents, hence form diverse group in this study. The categorizations primarily derived from fruit traits align with those previously identified in pepper landrace collections from Italy and Turkey [34]. However, in those studies, the plant descriptors elucidated varying percentages of the variance, ranging from 13.9% [6] to 62.4% [34–36]. Similar patterns have been observed in other Capsicum species and closely related horticultural crops, such as tomatoes [37–39]. The outcomes of this investigation hold significant results for plant breeders, providing insights into the development of cultivars that are not only nutritionally dense but also has high pungency—a combination that is desired by consumers within the food sector.

## Conclusion

We found high genetic diversity for agronomic traits and bioactive phytochemicals in Pakistani chilli pepper germplasm using phenotypic and HPLC assays. For improving agronomic traits genotypes are suggested as potential parents. The genotypes have high contents and are suggested as potential parents for improving nutritional value of Chilli pepper. The strong correlation between these two traits suggested that these traits can be considered for selection for simultaneous improvement.

## Data Availability

The datasets generated during and/or analysed during the current study are available from the corresponding author on reasonable request.

## Abbreviations

HPLC: High proficiency liquid chromatography
LSI: Least significant increase
LOD: Logarithm of odd
LOQ: Logarithm of quantitation
PCA: Principal component analysis
TFC: Total flavonoid contents
TPC: Total phenolic contents
ASCA: Ascorbic acid
PUN: Capsaicin contents
